# An AI-Based Solution for Denoising Fast-Acquisition [^18^F]FDG PET: Clinical Feasibility and Quantitative Assessment

**DOI:** 10.1007/s10278-025-01638-9

**Published:** 2025-08-28

**Authors:** Luísa C. Silva, Cláudia S. Constantino, Ricardo Teixeira, Joana C. Castanheira, Francisco P. M. Oliveira, Durval C. Costa

**Affiliations:** Present Address: Champalimaud Clinical Centre, Champalimaud Foundation, Av. Brasília, 1400-038 Lisbon, Portugal

**Keywords:** Positron emission tomography (PET), Fast scan, Deep learning, Denoising, Oncology, Clinical assessment

## Abstract

Benefits in patient comfort, efficiency, and sustainability can come from reducing positron emission tomography (PET) scan’s acquisition duration. This study assesses the clinical adequacy of restoring fast-acquisition ^18^F-fluorodeoxyglucose ([^18^F]FDG) PET to its standard-of-care image quality through deep-learning-based (DL) methods. Fast and standard whole-body [^18^F]FDG PET acquisitions of 117 oncological patients were included in the training and testing of three convolutional neural networks. The best-performing network during training was chosen for clinical evaluation on the test set (*N* = 25). Visual assessment and lesion detectability of the fast acquisitions, of 20 and 30 seconds per axial field of view (s/AFOV), with and without DL-based denoising, and of the local standard of care, of 70 s/AFOV, were performed by three experienced nuclear medicine physicians. Quantification was conducted globally (voxel-wise), in healthy organs and the reported lesions. Optimised Gaussian and non-local means filters served as benchmarks. Visual assessment revealed 20 and 30 s/AFOV with DL-based denoising to have similar image quality to the standard of care. Average lesion-based sensitivity and positive predictive value were 74% and 72%, respectively, for 20 s/AFOV + DL and 72% and 80% for 30 s/AFOV + DL. DL-based denoising displayed the highest voxel-wise agreement with the standard-of-care (*p* < 0.001). Liver and lungs in the DL-denoised images exhibited a higher signal-to-noise ratio than the standard of care. The median absolute maximum standardised uptake value deviation in the lesions was as low as 0.39 for 20 s/AFOV + DL and 0.30 for 30 s/AFOV + DL. The proposed DL-based method proved to be suitable for the restoration of fast-acquisition whole-body [^18^F]FDG PET, having resulted in images similar to the standard-of-care acquisitions. DL-based denoising outperformed standard benchmark methods.

**Supplementary Information:**

The online version contains supplementary material available at 10.1007/s10278-025-01638-9.

## Introduction

Ever since its first implementation for clinical evaluation in the 1990s, positron emission tomography (PET) has been establishing itself as a key imaging technique in oncology [1] and neurology [2], as well as other medical domains. The basis for PET is the administration of a radioisotope-labelled biological molecule, a radiopharmaceutical, which allows for the observation of the biological function in question. Nowadays, PET acquisition systems are hybrid, integrating an X-ray computed tomography (CT) module. Referral to a PET/CT scan, in oncology, usually arises if the clinician suspects the presence of active tumours, with a common radiopharmaceutical being fluorine-18-labelled fluorodeoxyglucose ([^18^F]FDG), an analog of glucose. Following diagnosis, PET is a tool for cancer staging, re-staging, prognosis assessment and treatment planning, and monitoring.

PET scans require the patient to lie still in the equipment for a long period, which is often uncomfortable and uneasy, and increases the susceptibility to motion artefacts. Other concerns raised are the exposure to radiation of patients and staff, as well as the regards for energy efficiency and rentability of the acquisition system. There is, therefore, a common interest in optimising this technique in terms of the patient’s comfort, radiological protection, and sustainability. A possible approach is to either reduce the PET acquisition duration or to reduce the administered activity. However, both lead to the deterioration of the resulting image.

In PET/CT imaging, various factors influence image quality. These include scanner specifications (such as sensitivity and spatial resolution), reconstruction technique, imaging protocol, and even patient habitus and demographics. In the context of this study, as posed before, the most relevant are the activity administered and the acquisition duration. Reducing the administered activity or the PET acquisition duration results in an image with higher prominence of noise, as fewer counts are detected in either case. This noise pattern is considered to follow a mixed Gaussian-Poisson model [3], making the image difficult to restore. Nevertheless, it has been shown that it is feasible to reduce the PET acquisition duration without affecting the clinical value of the image [4]. This trade-off between acquisition time and image quality is one of the main challenges to address.

In recent years, artificial intelligence (AI) and, particularly, machine learning (ML) have been found to have numerous applications in healthcare [5, 6]. These applications span multiple data modalities and involve a range of tasks—most commonly classification and regression (e.g. disease diagnosis [7, 8] and risk prediction [9]), but also, increasingly, segmentation [10] and various forms of image reconstruction or enhancement [11], particularly in medical imaging. Deep learning (DL) is an ML technique that allows a system to build and learn concepts, with progressive levels of complexity and abstraction that attempts to mimic the human brain. Apropos of PET/CT, it has been used to simulate, reconstruct, or enhance images, among other tasks, with promising clinical applications [12]. These methods have the potential to make it feasible to go beyond the clinically suitable acquisition duration by restoring the otherwise lost information. Some studies have already started to explore the potential of DL-based denoising/enhancement of low-activity/fast-acquisition PET, either by the clinical validation of commercially available software [13–16] or by the development and validation of in-house solutions [17–21]. Nonetheless, the optimal compromise between the reduction in acquisition duration and the detection of enough counts without losing clinically relevant information is not well-established. This is, the question of how low we can go in acquisition duration while still detecting enough signal to capture all [^18^F]FDG-avid lesions is left unanswered. Most studies [13, 16, 18, 19, 21] contemplate a reduction in acquisition duration from 45 s to 1 min per axial field of view (AFOV), which can be further reduced, exploiting DL-based methods to their full potential.

This study aims to explore the potential of DL-based denoising of images from fast-acquisition whole-body [^18^F]FDG PET scans by assessing qualitatively and quantitatively the enhanced images in comparison to those of standard clinical practice. Additionally, conclusions regarding the optimal fast-acquisition duration will be drawn by the clinical assessment of different acquisition durations.

## Materials and Methods

### Patient Dataset

This retrospective single-centre study included a random sample of 117 patients with various types of primary cancer, referred for clinical whole-body [^18^F]FDG PET/CT. One PET/CT study per patient was included. The acquisitions were performed in a Philips Vereos Digital PET/CT scanner, approximately 60 min post-injection, and with an acquisition duration of 70 s/AFOV. These scans, acquired following the local standard-of-care clinical protocol, served as the reference in this study. Table 1 displays the demographic characteristics of the patients included in the dataset, along with the average injected activity. All remaining clinical data was anonymised. Ethical approval was obtained from the institutional review board.

**Table 1:** Demographic and PET-related characteristics of the patients/studies included in the dataset

Parameter	Total (*N* = 117)	Train set (*N* = 92)	Test set (*N* = 25)
Gender (M/F)	47%/53%	50%/50%	36%/64%
Age [years]	65 ± 11	65 ± 11	63 ± 10
BMI [kg/m^2^]	26 ± 5	26 ± 4	28 ± 5
Injected activity [MBq/kg]	3.4 ± 0.2	3.4 ± 0.2	3.2 ± 0.3

*BMI* body mass index

Besides the standard 70 s/AFOV acquisition (PET70), the PET raw data was reconstructed with different acquisition durations: 10, 15, 20, 25, and 30 s/AFOV. The reconstructions fulfil the European Association of Nuclear Medicine Research Ltd. (EARL) ^18^F standards 1 accreditation [22]. Voxel size was 4 × 4 × 4 mm^3^ for all reconstructions.

### Deep-Learning-Based Approaches

**Fig. 1:**
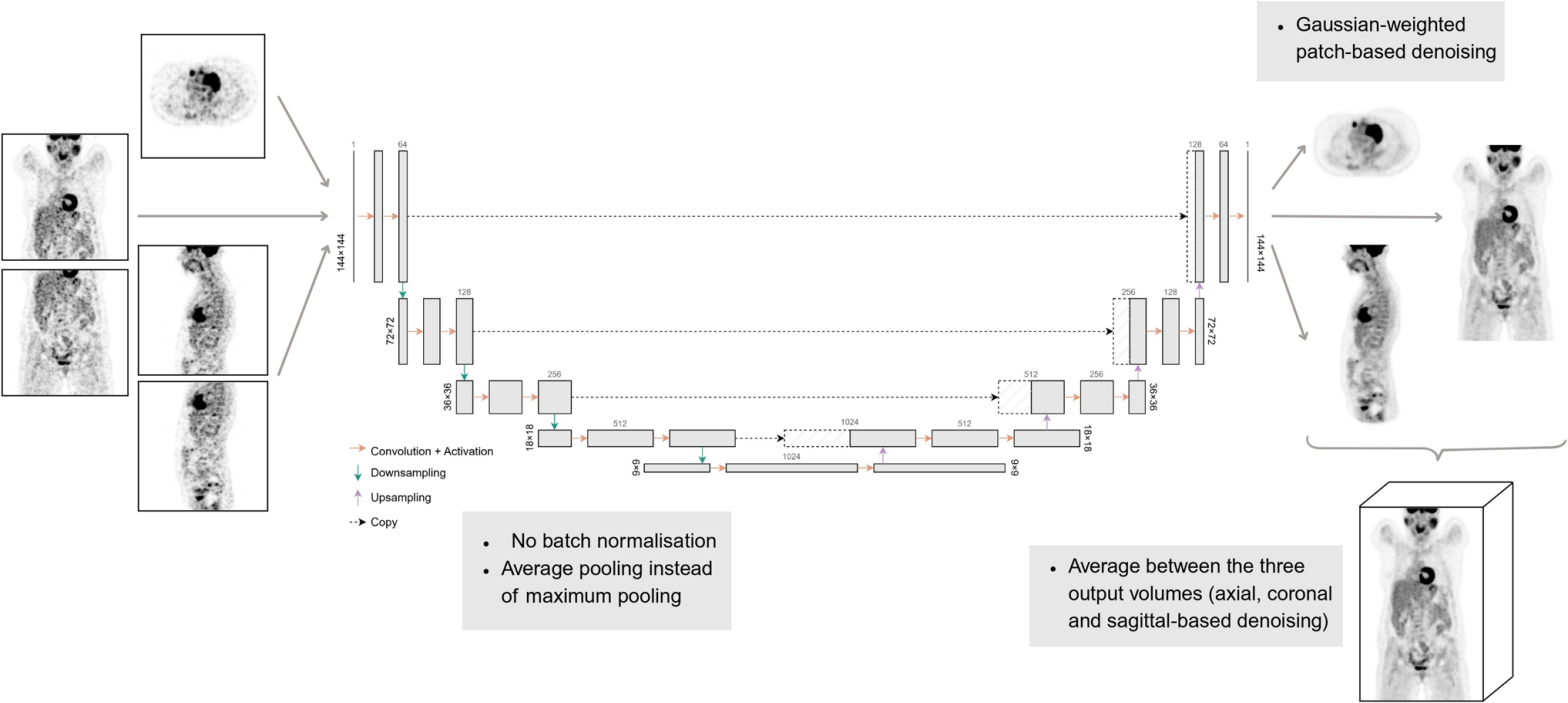
Schematic of the denoising process of a given volume through the 1-channel 2.5D U-Net model

The convolutional neural networks (CNNs) explored include those most seen in the literature—a “conventional” three-channel 2.5D U-Net [13–16, 21] of input each axial plane and its adjacent (immediately prior and successive) slices, and a 3D U-Net [18] of input 3D patches extracted from the whole volume—and a different 2.5D approach—a one-channel 2.5D U-Net of input a 2D image of any of the three anatomical planes (coronal, sagittal and axial). Alterations to the original U-Net [23] were performed, as the denoising task differs significantly from the segmentation task (for which the U-Net was developed). Average pooling was chosen over maximum pooling. Batch normalisation was not included. He et al. [24] weight initialisation was used. A schematic of how each volume is denoised for the one-channel 2.5D U-Net is shown in Fig. 1. Network architectures are shown in Figs. S1–S3 of the Supplementary Material. The networks were trained for 1000 epochs, with a starting learning rate of 0.001. Voxel-wise mean squared error (MSE) was used as the loss function. When training each CNN, model selection was based on the absolute validation loss minimum.

The patient dataset (*N* = 117) was randomly split: 80% (*N* = 92) for training and validation and 20% (*N* = 25) for testing. Table 1 reports the characteristics of these data partitions. To provide broader variability in noise level during training, acquisition durations of 10, 15, 20, 25, and 30 s/AFOV were included. Data augmentation included rotation, anisotropic scaling, and translation. Testing comprised the 20- and 30-s/AFOV acquisitions (PET20 and PET30, respectively).

### Benchmark Denoising Methods

One of the most common denoising techniques in medical imaging is Gaussian filtering, given its simplicity and efficiency in smoothing images [25]. However, its blurring effect is at times detrimental. Buades, Coll, and Morel [26] addressed this problem by introducing the non-local means (NLM) algorithm for image denoising. Both above-mentioned denoising methods were employed as benchmarks. Both filters’ parameters were optimised through MSE minimisation. Implementation and optimisation methodology are described in the Supplementary Material.

### Qualitative Analysis

Three experienced (> 10 years) nuclear medicine physicians examined independently the PET and respective CT images of the 25 patients of the test set for global visual assessment and lesion detection, in blinded and randomised readings, using 3D Slicer (www.slicer.org). The readers were blinded not only to the study type (staging, restaging, treatment response, etc.) but also to the patient’s clinical background.

Visual assessment was performed for reference images, fast acquisitions with no post-processing (PET20 and PET30), and respective fast acquisitions with DL-based denoising. A 5-point Likert scale was employed to categorise image quality relative to routine/standard clinical practice: 1, extremely lower; 2, lower; 3, equivalent; 4, higher; 5, extremely higher.

For lesion detectability, only the reference and DL-denoised images were included. PET20-DL and PET30-DL, along with the respective reference images, were assessed independently with an adequate interval distancing each set in time. The evaluations were carried out in several sessions, in such a way that no patient was duplicated in each session. The physician was asked to identify the suspected [^18^F]FDG-avid lesions or regions considered being of abnormal [^18^F]FDG uptake. A lesion candidate was considered only if two or more physicians identified it (majority basis). The majority-based lesion identification performed on the reference PET70 images served as the ground truth for the subsequent analysis. Lesion-based sensitivity and positive predictive value (PPV) were then assessed. As the reference images were assessed twice (alongside PET20-DL and PET30-DL), the intra-reader variability in evaluating the reference set was assessed. The first reading of PET70 will be referred to as PET70-1 and the second as PET70-2.

### Quantitative Analysis

A voxel-wise analysis between non-denoised and denoised images against the respective references was performed in terms of MSE, structural similarity index measure (SSIM) [27], and intra-class correlation coefficient (ICC) for absolute agreement [28].

Regional analysis in signal-to-noise ratio (SNR) and mean standardised uptake value (SUV_mean_) was conducted in regions of expected uptake uniformity in the liver and lungs. The delineation of these regions was done manually in the reference images.

For lesion quantification, semi-automatic Bayesian segmentation [29, 30] was applied independently to each set of test images from the majority-based masks outlined by the physicians. The ground truth segmentation corresponded to the intersection between the two readings of PET70. A given lesion was only considered for quantification if its segmentation resulted in a metabolic tumour volume (MTV) equal or superior to 4 voxels (0.256 cm^3^). Feature extraction included SUV_max_, SUV_mean_, SUV_peak_, total lesion glycolysis (TLG) and MTV.

Statistical significance was assessed through the Friedman test for repeated measures and the Wilcoxon signed-rank test (post hoc). A significance level of 5% was established. Multiple comparison correction was not performed.

## Results

### Deep-Learning-Based Approach Selection

The performance of the three CNNs tested is displayed in Tables S1–S4 of the Supplementary Material. CNN selection was based on a custom score that weighs in both voxel-wise and regional quantification measures, as per Tables S5 and S6 of the Supplementary Material. The best-performing network was the one-channel 2.5D U-Net (of input each coronal, sagittal, and axial planes of the volume). The 20- and 30-s/AFOV test sets denoised through the selected CNN model will be referred to as PET20-DL and PET30-DL, respectively.

### Benchmark Optimisation

From within the training set, the optimal Gaussian filter full width at half maximum obtained was 5 mm. The optimisation results are shown in Fig. S4 of the Supplementary Material.

The optimal NLM filter was the one of maximum patch distance 3 (7 × 7 × 7 neighbourhood) and weight-decay constant (*h*) 0.3, given a kernel size of 3 × 3 × 3 voxels. The optimisation results are shown in Fig. S5 of the Supplementary Material.

Both filters were applied to the fast-acquisition test set, and the resulting images (denoised through the Gaussian filter—GF-denoised—and through the NLM filter—NLM-denoised) were used as benchmarks. The GF-denoised and NLM-denoised 20- and 30-s/AFOV-based test sets will be referred to as PET20-GF and PET20-NLM, and PET30-GF and PET30-NLM, respectively.

**Fig. 2:**
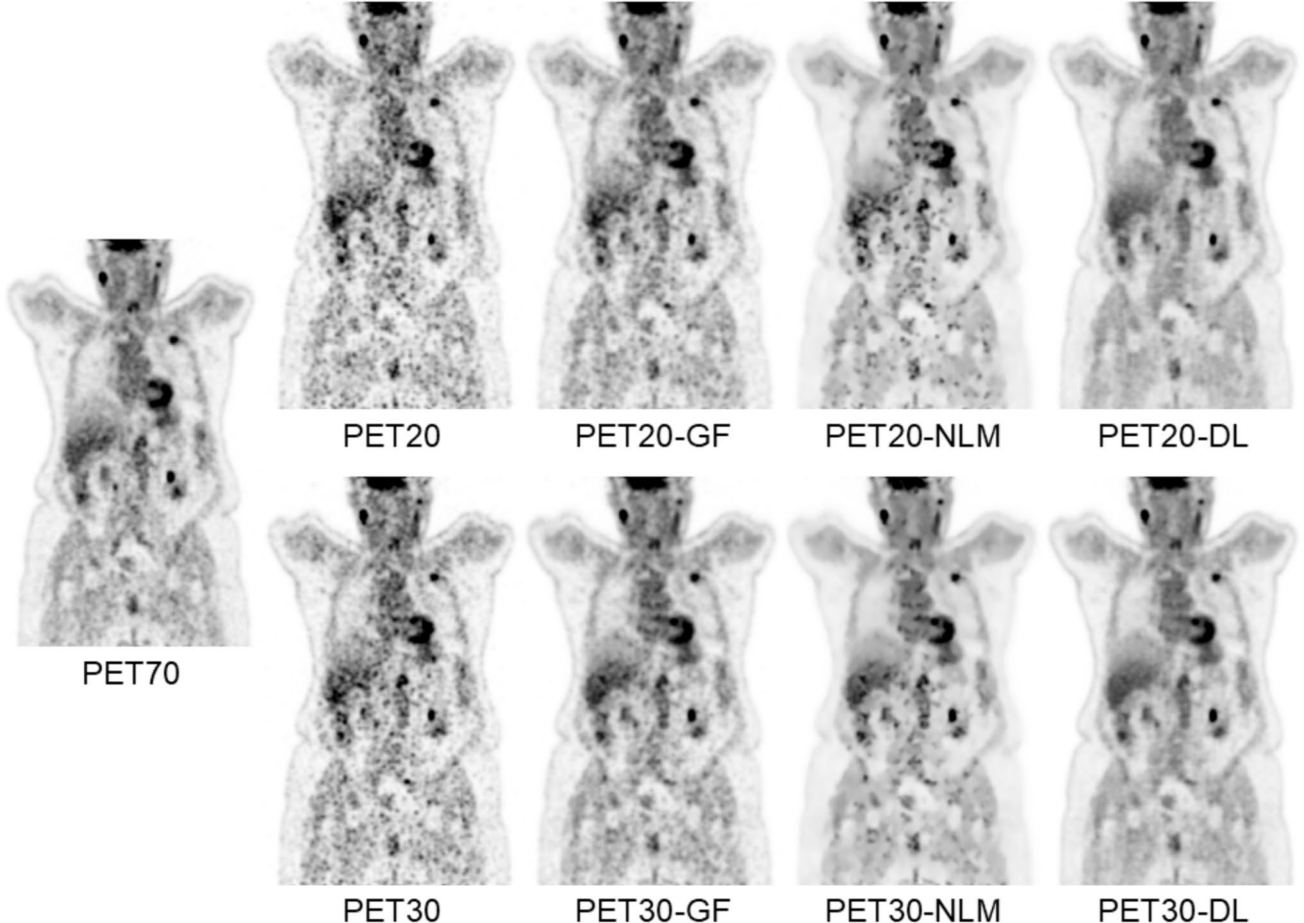
Coronal views of a patient from the test set: PET70 (70-s/AFOV reference acquisition), PET20 and PET30 (non-denoised fast acquisitions with 20 and 30 s/AFOV, respectively), and the denoising results for the three methods considered—Gaussian filter (GF), non-local means (NLM) filter, and deep-learning-based denoising (DL)

Figure 2 displays coronal views of PET70, PET20, and PET30 of a subject from the test set, and all the respective denoising results (DL, GF, and NLM).

### Voxel-wise Quantification

Table 2 displays the voxel-wise quantification results. Statistically significant differences were observed in MSE, ICC, and SSIM between the original and the denoised sets (*p* < 0.001). A statistically significant improvement in favour of DL-based denoising was observed for the three measures (*p* < 0.001), i.e., a decrease in MSE and an increase in both SSIM and ICC were observed for DL-based denoising, compared with the other image sets.

**Table 2:** Voxel-wise analysis results. Average MSE, SSIM and ICC (± standard deviation) between the image sets and respective reference

Image set	MSE [× 10^−3^ g^2^/mL^2^]	SSIM	ICC
PET20	15 ± 7	0.91 ± 0.03	0.979 ± 0.007
PET20-GF	12 ± 6	0.92 ± 0.02	0.983 ± 0.006
PET20-NLM	13 ± 6	0.89 ± 0.03	0.982 ± 0.007
PET20-DL	8 ± 3	0.94 ± 0.02	0.989 ± 0.004
Friedman test *p*-value	< 0.001*	< 0.001*	< 0.001*
PET30	9 ± 4	0.94 ± 0.02	0.987 ± 0.004
PET30-GF	8 ± 4	0.94 ± 0.02	0.988 ± 0.004
PET30-NLM	9 ± 4	0.89 ± 0.03	0.987 ± 0.004
PET30-DL	6 ± 3	0.95 ± 0.02	0.992 ± 0.003
Friedman test *p*-value	< 0.001*	< 0.001*	< 0.001*

*Post hoc analysis through the Wilcoxon signed-rank test showed statistically significant differences between each pair of image sets

*MSE* mean squared error, *SSIM* structural similarity index measure, *ICC* intraclass correlation coefficient, *GF* Gaussian filter, *NLM* non-local means filter, *DL* deep-learning-based denoising

### Regional Signal-to-Noise Ratio Assessment

Table 3 showcases the results of the quantification in regions of expected uptake uniformity in the liver and lungs. DL denoising exhibits a consistent increase in SNR relatively to the reference, contrarily to the other image sets. Regarding SUV_mean_, an average variation of no more than 5% was observed for the DL-denoised sets, with similar or larger variations for the remaining sets of images.

**Table 3:** Normal-uptake organ quantification analysis: average relative difference (± standard deviation) to the reference in terms of SNR and SUV_mean_, from within the test sets—original (not denoised, 20 and 30 s/AFOV acquisitions) and denoised through the three methods studied

ROI	Liver		Lungs	
Image set	ΔSNR [%]	ΔSUV_mean_ [%]	ΔSNR [%]	ΔSUV_mean_ [%]
PET20	− 44 ± 10	− 1 ± 6	− 33 ± 12	− 2 ± 8
PET20-GF	− 24 ± 15	− 1 ± 6	− 14 ± 15	− 2 ± 7
PET20-NLM	+ 33 ± 47	− 2 ± 6	+ 112 ± 57	− 1 ± 6
PET20-DL	+ 104 ± 44	+ 1 ± 4	+ 49 ± 33	+ 3 ± 6
PET30	− 32 ± 12	0 ± 4	− 21 ± 13	− 1 ± 6
PET30-GF	− 7 ± 18	0 ± 4	0 ± 17	0 ± 5
PET30-NLM	+ 104 ± 74	− 1 ± 3	+ 127 ± 57	+ 2 ± 5
PET30-DL	+ 112 ± 44	+ 1 ± 3	+ 48 ± 30	+ 2 ± 5

*ROI* region of interest, *SNR* signal-to-noise ratio, *ΔSNR* signal-to-noise ratio variation, *SUV*_*mean*_ mean standardised uptake value, *ΔSUV*_*mean*_ SUV_mean_ variation, *GF* Gaussian filter, *NLM* non-local means filter, *DL* deep-learning-based denoising

### Qualitative Analysis

**Fig. 3:**
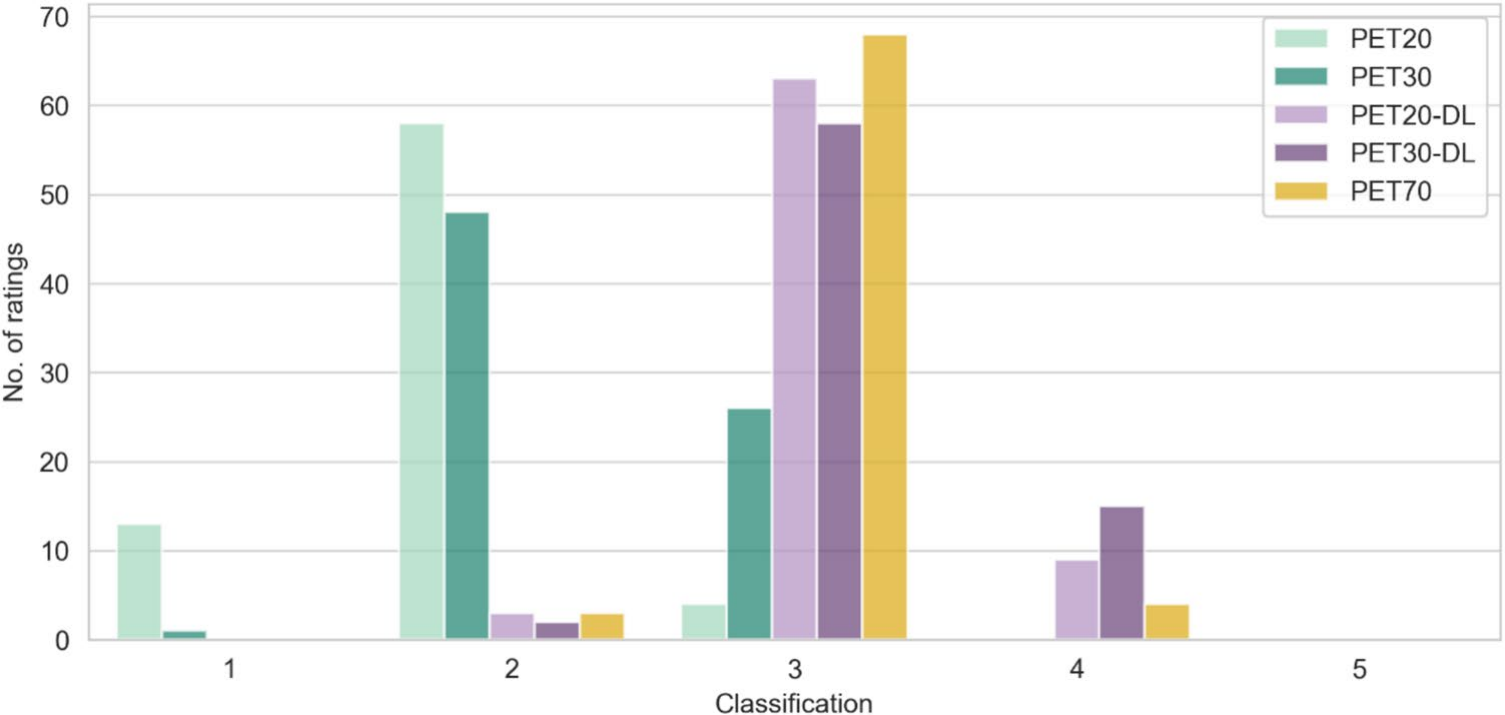
Distribution of image quality relative to routine/standard clinical practice (70 s/AFOV)—PET70—(5-point Likert scale) for the evaluated image sets, 20 and 30 s/AFOV, with and without DL-based post-processing—PET20, PET30, PET20-DL, PET30-DL—in total (ratings by all readers)

The average classifications from within the 5-point Likert scale to categorise the images’ quality relative to routine/standard clinical practice were 1.9 ± 0.5 for PET20, 2.3 ± 0.5 for PET30, 3.1 ± 0.4 for PET20-DL, 3.2 ± 0.4 for PET30-DL, and 3.0 ± 0.3 for PET70 (Fig. 3). PET20 and PET30 were excluded from the subsequent analysis in terms of lesion detectability as they were considered, on average, not to have appropriate image quality for clinical use.

A total of 125 suspected lesions were identified in PET70-1 and 111 in PET70-2, against 121 in PET20-DL and 106 in PET30-DL. The results for lesion detectability are displayed in Table 4. The results are satisfactory in all cases. As expected, slightly higher values for sensitivity and PPV are observed when comparing the two readings of PET70, as the object images are the same.

**Table 4:** Lesion detectability in terms of sensitivity and positive predictive value, for each reviewed set (20 and 30 s/AFOV acquisitions), regarding the first reading of PET70, PET70-1, or the second, PET70-2

Set	Reference	Sensitivity	PPV
PET20-DL	PET70-1	0.70	0.73
PET30-DL		0.68	0.80
PET70-2		0.80	0.90
PET20-DL	PET70-2	0.77	0.71
PET30-DL		0.76	0.79
PET70-1		0.86	0.77

*PPV* positive predictive value, *DL* deep-learning-based denoising

**Fig. 4:**
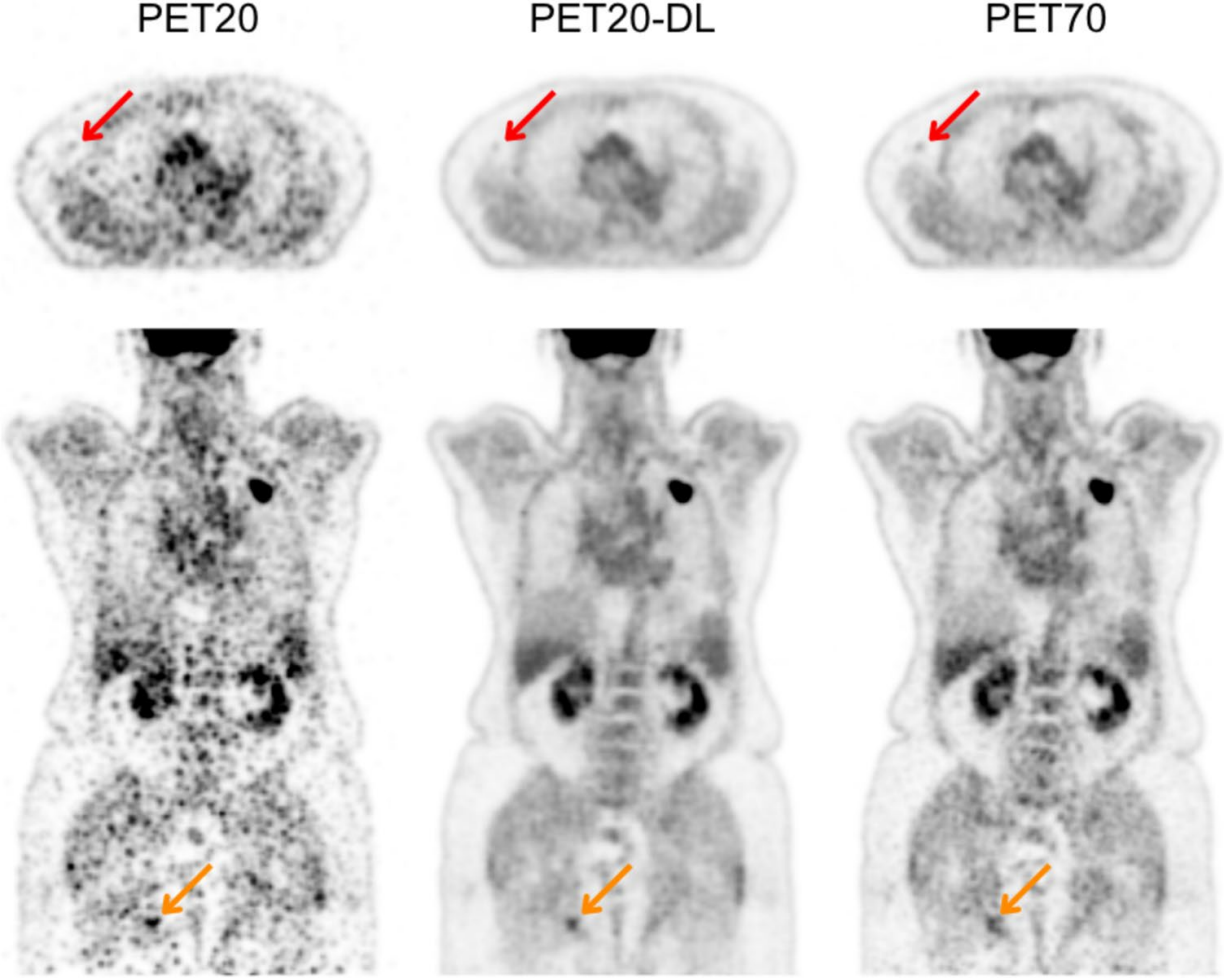
[^18^F]FDG PET acquisition of a patient from the test set, for which a classification of 1 (extremely lower) in visual image quality relative to the standard of care was given by 2/3 readers to the 20 s/AFOV acquisition (PET20). A classification of 4 (higher image quality) was given by 1/3 readers to PET20 + DL-based denoising (with the remaining giving a classification of 3, meaning similar image quality to standard of care). A classification of 3 was given by 3/3 readers to the 70-s/AFOV reference acquisition (PET70). Red arrows refer to a false negative—a lesion missed in PET20-DL but detected in PET70. Orange arrows refer to a false positive—a “lesion” detected in PET20-DL but “missed” in PET70

Figure 4 exhibits a study of the PET20 test set in which a false negative and a false positive were found. The PET20 image was classified as having extremely low image quality relative to standard clinical practice (score of 1) by two out of the three readers. The respective PET20-DL image was classified as having higher image quality than standard clinical practice (score of 4) by one reader.

### Lesion Quantification

**Fig. 5:**
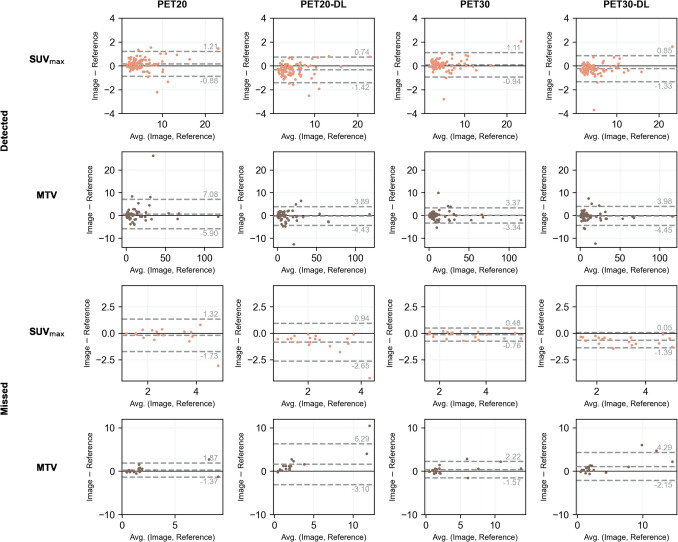
Bland–Altman plots for maximum SUV and metabolic tumour volume of the both groups of lesions—those detected and those missed in the DL-denoised sets, for both fast-acquisition durations (20 and 30 s/AFOV—PET20 and PET30, respectively)

Table 5 displays the median absolute deviations in the features of the two groups of lesions: those correctly detected and those missed in PET20-DL and PET30-DL. The denoised sets display small differences relative to the reference, although slightly higher than those observed for the non-denoised, fast acquisitions. For the detected lesions in PET20-DL, average SUV_max_ and MTV in PET70 were 4.96 and 9.3 cm^3^, respectively. For those detected in PET30-DL, average SUV_max_ and MTV in PET70 were 5.0 and 10.2 cm^3^, respectively. For the missed lesions in PET20-DL, average SUV_max_ and MTV in PET70 were 2.6 and 1.8 cm^3^, respectively. For those missed in PET30-DL, average SUV_max_ and MTV in PET70 were 2.9 and 2.8 cm^3^, respectively. Figure 5 displays the SUV_max_ and MTV Bland–Altman plots for the detected and missed lesions in PET20-DL and PET30-DL.

**Table 5:** Median absolute deviation in the features (SUV_max_, SUV_mean_, SUV_peak_, TLG, and MTV) of the two groups of lesions: those detected in both PET20-DL/PET30-DL and PET70 and those missed in PET20-DL/PET30-DL

Image set	Group	|ΔSUV_max_| [g/mL]	|ΔSUV_mean_| [g/mL]	|ΔSUV_peak_| [g/mL]	|ΔTLG| [g]	|ΔMTV| [cm^3^]
PET20	Detected (*N* = 92)	0.31	0.17	0.12	1.22	0.64
PET20-GF		0.47	0.23	0.15	1.27	0.70
PET20-NLM		0.40	0.27	0.20	1.57	0.83
PET20-DL		0.37	0.22	0.16	1.33	0.64
Friedman test* p*-value		0.17	0.06	< 0.05	0.26	< 0.05
PET20	Missed (*N* = 21)	0.27	0.17	0.10	0.62	0.19
PET20-GF		0.30	0.26	0.08	0.65	0.45
PET20-NLM		0.75	0.44	0.10	0.71	0.45
PET20-DL		0.55	0.43	0.10	0.66	0.64
Friedman test* p*-value		< 0.05	< 0.05	0.54	< 0.05	< 0.05
PET30	Detected (*N* = 86)	0.24	0.14	0.13	0.96	0.51
PET30-GF		0.56	0.24	0.14	1.00	0.67
PET30-NLM		0.28	0.19	0.18	1.34	0.70
PET30-DL		0.29	0.17	0.14	1.17	0.61
Friedman test* p*-value		< 0.05	< 0.05	0.14	0.34	< 0.05
PET30	Missed (*N* = 22)	0.14	0.13	0.12	0.62	0.42
PET30-GF		0.36	0.26	0.12	0.63	0.48
PET30-NLM		0.62	0.48	0.24	0.86	0.99
PET30-DL		0.50	0.35	0.17	0.68	0.54
Friedman test* p*-value		< 0.05	< 0.05	0.09	0.15	0.21

*SUV*, standardised uptake value; *SUV*_*max*_, maximum SUV; *SUV*_*mean*_, mean SUV; *SUV*_*peak*_, peak SUV; *SNR*, signal-to-noise ratio; *TLG*, total lesion glycolysis; *MTV*, metabolic tumour volume; |Δ $x$|, absolute variation of variable $x$ (either SUV_max_, SUV_mean_, SUV_peak_, TLG or MTV); *GF*, Gaussian filter; *NLM*, non-local means filter; *DL*, deep-learning-based denoising

Only one-third of the 18 missed lesions in PET20-DL were not detected at all. At least one physician detected the remaining. For PET30-DL, none of the 22 considered-missed lesions were completely missed, with all lesions detected by, at least, one physician.

## Discussion

This study aimed to explore the feasibility and practicability of reducing the acquisition duration of whole-body [^18^F]FDG PET through DL-based denoising. Our denoising method was based on the U-Net architecture [23]. Three different versions of this network were explored. Although only the best-performing one was considered for subsequent qualitative analysis, all solutions proved to be viable for this study’s purpose. Despite their popularity, generative adversarial networks (GANs) were not considered in this study given the conceptual limitation in controlling the truthfulness of the output.

The average injected activity for the studies included in the dataset was 3.4 ± 0.2 MBq/kg, which complies with the guidelines for PET/CT imaging [22]. Similar studies reveal around 2–3 min/AFOV to be the acquisition duration in standard clinical practice [13, 16–18, 31]. Our 70 s/AFOV reference acquisitions are faster than this standard. This comes as a result of a local protocol optimisation for improving patient comfort and efficiency. The tested fast acquisitions were those of 20 and 30 s/AFOV, which is lower than those considered in most similar studies. Mehranian et al. [18] employed durations ranging from approximately 40 to 110 s/AFOV for training and testing a 3D U-Net. Tsuchiya et al. [19] included 2-min/bed scans to test a 2.5D residual neural network. Bonardel et al. [13] and Weyts et al. [16] assessed the performance of an already-commercialised solution, SubtlePET, on acquisitions of duration ranging from 30 to 60 s/AFOV. The quantitative analyses performed in the reviewed studies generally show DL-based solutions to be able to restore the fast acquisitions’ quantitative parameters. Hosch et al. [17] opted for a bolder reduction, employing acquisitions of approximately 5 s/AFOV, to test a GAN-based solution. Although the AI-based solution demonstrated a fair performance in quantitative measures, the qualitative assessment suggested that longer acquisitions were needed for generalised clinical use. Accounting for this, this study contemplated 20 and 30 s/AFOV as the proposed fast-acquisition durations.

The clinical qualitative assessment, an absolute consideration in establishing the feasibility of the proposed methods, was performed retrospectively on anonymised data, and contemplated lesion detectability and visual image quality. In clinical practice, more than lesion detectability, it is important to observe the clinical outcome, as detecting or not a given lesion among others may influence the subsequent decisions. Therefore, the applicability of these methods must be investigated in conditions closer to the normal clinical setting.

Voxel-wise quantification favoured DL-based denoising. To corroborate these results, and specifically for clinical application, image quality relative to standard clinical practice was assessed. The physicians considered the DL-denoised fast acquisitions to have similar image quality to the reference.

Regarding healthy organ quantification, PET20 and PET30 displayed a significant decrease in SNR in both the liver and lungs. DL-based denoising revealed an increased SNR in the liver and lungs, in comparison with the reference. Even though an increase in SNR usually translates into better image quality, an excessively smoothed image would exhibit an increased SNR while possibly having lost clinically relevant information. The assessment of image quality during qualitative analysis allowed this aspect to be taken into account.

Lesion detectability was assessed not only for PET20-DL and PET30-DL but also for PET70, which was evaluated independently twice. The variability between these two independent readings of the reference set supplies a baseline for expected detectability. Overall detectability was expressed in terms of sensitivity. This analysis revealed lesion detectability to be similar between PET20-DL and PET30-DL, and slightly inferior than the normal variability in different readings of the same set. PPV was higher for PET30-DL than for PET20-DL and similar to the normal variability. The overall results obtained in this study align with the view of Hosch et al. [17], proposing the implementation of these methods in cases where single-lesion detection is not the main concern.

Lesion quantification did not reveal any clinically relevant discrepancies in SUV_max_. The missed lesions in PET20-DL and PET30-DL corresponded to low values of SUV_max_ (SUV_max_ < 5). These findings suggest that a fast acquisition protocol may not be ideal for cases of initial staging of metastatic disease. Although PET20-DL and PET30-DL displayed similar lesion detectability, quantification of the missed lesion sets reveals higher discrepancies for PET20-DL, as PET30-DL exhibits narrower 95% agreement intervals for SUV_max_ and MTV (Fig. 5). Missing a lesion with SUV_max_ and MTV equivalent to the reference points more towards the inevitable intrareader variability than to post-processing-induced loss of contrast. This is further corroborated by no lesion being completely missed in PET30-DL (at least one out of the three readers detected it).

The 10-second difference in acquisition duration from 30 to 20 s/AFOV does not seem to justify the loss in clinical value. The PET20-DL studies showed a higher tendency to display false negatives and false positives (as seen in Fig. 4)—the higher prominence of noise makes it harder for the model to distinguish between noise peaks and small lesions, as well as to disregard some higher-amplitude noise peaks as actual noise.

Although DL-based solutions have been at the core of medical image processing research, they require complex methodology and sometimes not-easily-attainable resources. It is, therefore, important to establish a benefit in performance, which was done by comparing against Gaussian filtering and adaptive non-local means filtering. Performance-wise, both methods fell short from the DL-based solution.

This study comprises some limitations that must be considered. Firstly, 117 volumetric acquisitions were included for training and testing the CNNs, which is suboptimal. Nonetheless, this is within the range of the number of images included in similar studies [5, 8, 11]. These acquisitions were performed on the same PET/CT equipment, meaning the dataset lacks inter-scanner variability. Notwithstanding, for an in-house solution (intended for local use), it is sufficient for the data only to be locally representative. Other than these, there is the already-mentioned limitation of employing 70 s/AFOV as the reference acquisition duration. Employing longer acquisitions in the training would provide a fairer comparison between the DL-denoised short acquisitions and our local standard of care. Lastly, the fast acquisitions employed for training and testing the CNNs were simulated by cropping the raw data of the standard-of-care long acquisition to the desired time window. However, real-world fast acquisitions are not expected to differ significantly from those simulated in this study, neither in terms of noise pattern and prominence nor in terms of radiopharmaceutical biodistribution. Low-count PET images obtained by reducing administered activity or by delayed acquisition are expected to have slightly higher background noise.

As it applies to other software solutions without proper certification from the regulatory authorities for clinical usage, the computational solution here proposed for denoising [^18^F]FDG PET imaging should only be used as a research tool.

Future steps for further validation of this method in real-world clinical application should focus on endorsing the best compromise between acquisition duration and image quality—around 30 s/AFOV, as per this study’s findings—and on the clinical outcome, as adequate quantitative agreement has been established. Additionally, real-world fast acquisitions will be needed for validation of the methods before clinical translation.

## Conclusion

The proposed reduction in whole-body [^18^F]FDG PET acquisition duration (to 30 s/AFOV) followed by DL-based denoising resulted in images similar to the local standard-of-care acquisitions.

Lesion detectability decreased slightly compared with the standard of care, although lesion quantification displayed similar quantitative parameters. 30 s/AFOV is the best compromise between acquisition duration reduction and image quality, allowing for a good agreement in image quality with the standard-of-care acquisitions, as well as satisfactory clinical assessment in visual image quality and lesion detectability. DL-based denoising outperformed standard methods.

The applicability of the proposed methods must be investigated prospectively in different clinical settings, focusing more on clinical outcomes than on lesion detectability. The conditions in which a fast-acquisition protocol may be applied must be investigated.

## Supplementary Information

Below is the link to the electronic supplementary material.

ESM 1 (DOCX 213 KB)

## Data Availability

Models, network implementation, and related scripts are available at https://github.com/NM-Radiopharmacology/FastPETDenoising**.**
