# Supplementary material for: An AI-Based Solution for Denoising Fast-Acquisition [18F]FDG PET: Clinical Feasibility and Quantitative Assessment
Source: J Imaging Inform Med. 2025 Aug 28;39(3):2582–92. doi: 10.1007/s10278-025-01638-9 (PMC13230395; doi:10.1007/s10278-025-01638-9)
Supplement: Supplementary file 1 — (DOCX 213 KB) [file 10278_2025_1638_MOESM1_ESM.docx]

An AI-based Solution for Denoising Fast-Acquisition [^18^F]FDG PET:

Clinical Feasibility and Quantitative Assessment

**Supplementary Material**

# Materials and Methods

## Deep-Learning-Based Approaches


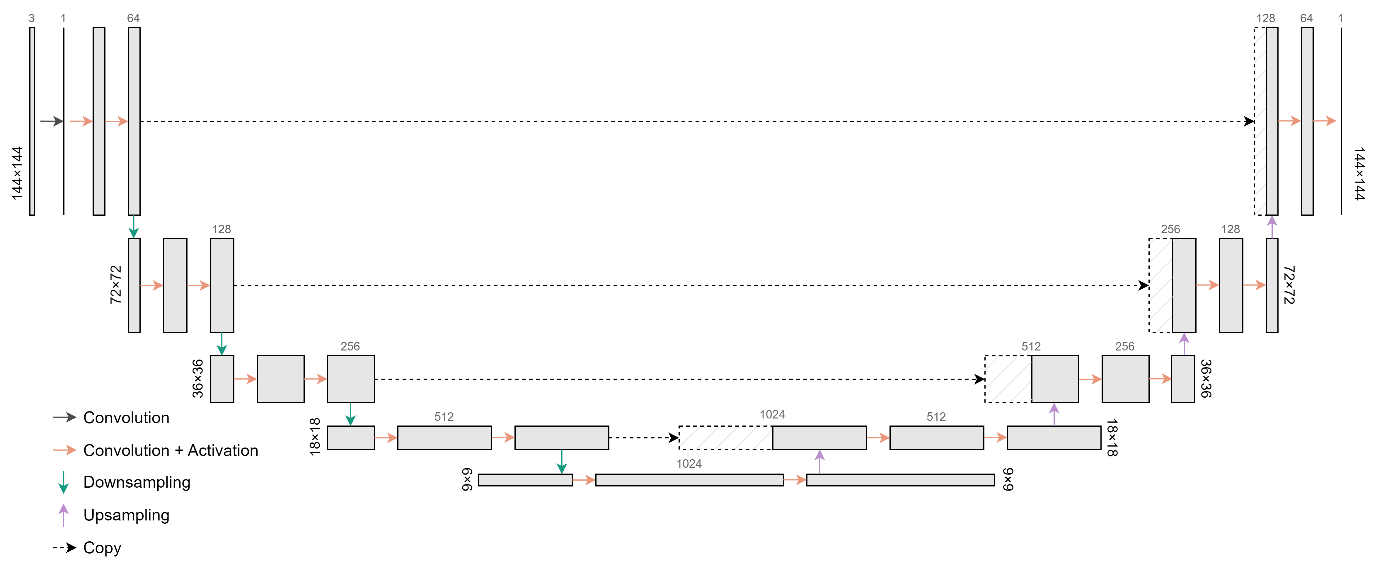


Figure S1 – Schematic of the architecture of a 4-block 2.5D U-Net. The network’s input is a 3-channel 144×144-pixel image of each axial slice and its adjacent (immediately prior and successive) slices.


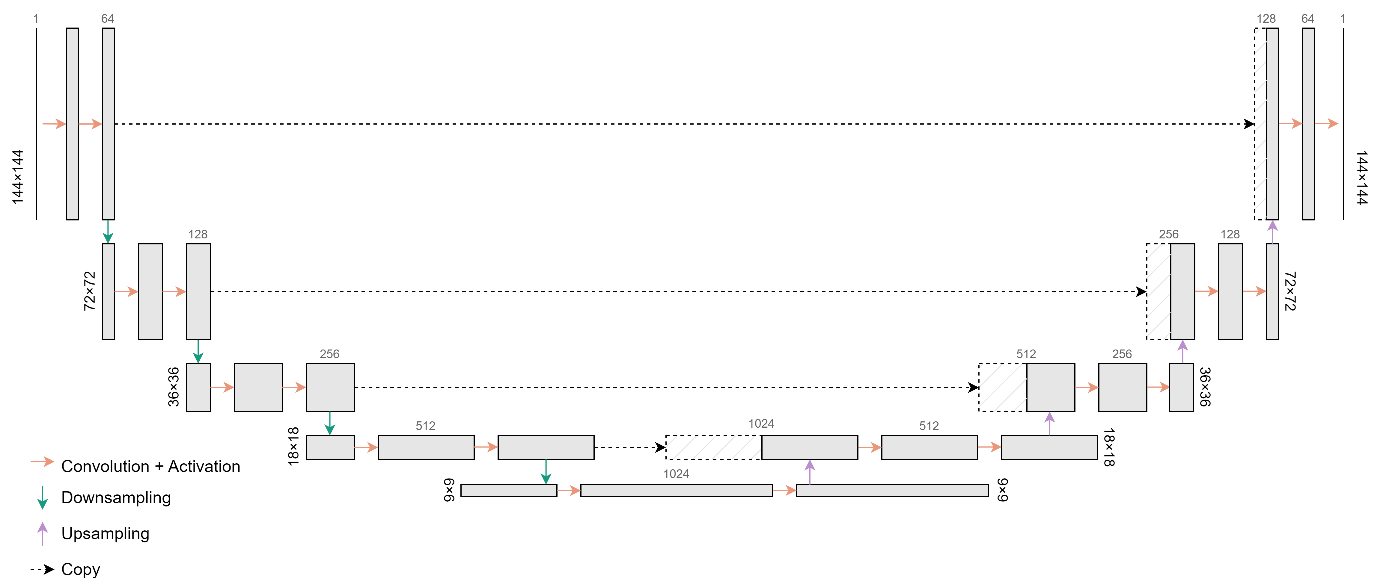


Figure S2 - Schematic of the architecture of a 4-block 2.5D U-Net. The network’s input is a 2D 144×144-pixel patch, of either the axial, coronal or sagittal planes.


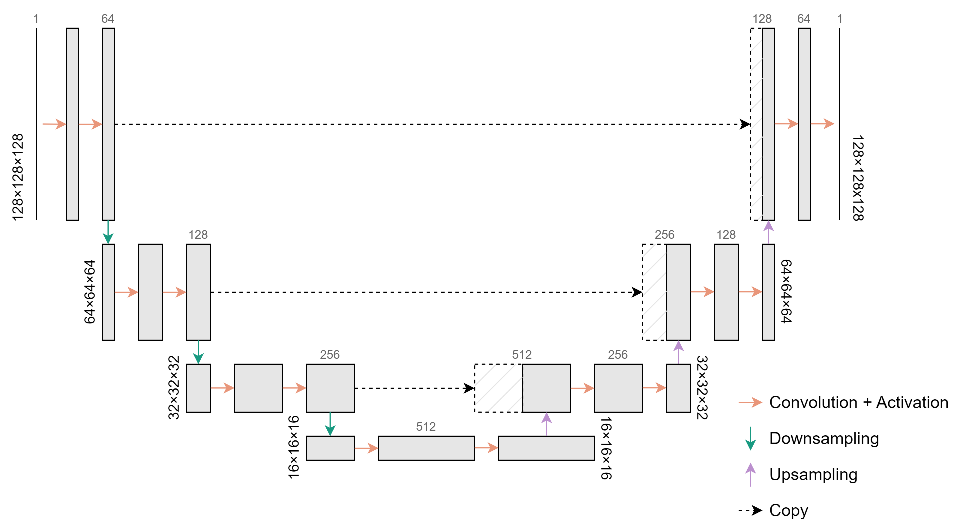


Figure S3 - Schematic of the architecture of a 3-block 3D U-Net. The network’s input is a 3D 128×128×128 vx patch.

## Benchmark Denoising Methods

Python’s open-source library scipy [18] provided an implementation of the Gaussian filter. The filter was optimised in terms of full width at half maximum (FWHM), through the minimisation of MSE (relatively to the reference images), in the training set. The filter widths tested for each scan duration ranged from 1 to 10 mm. The different filters were obtained by applying successive increments of 0.5 mm to the starting 1 mm filter FWHM. The NLM filter was implemented in C++ [19]. The filter’s parameters were also optimised in the training set through the MSE minimisation between denoised and reference images. The filter’s kernel size was 3×3×3 voxels, the maximum patch distances tested were 3 and 5 (corresponding to neighbourhoods of 7×7×7 and 11×11×11 voxels, respectively), and the tested filter’s thresholds (*h*) ranged from 0.10 to 0.40 with 0.05 increments.

# Results

## Deep-Learning-Based Approach Selection

Table S1 – Voxel-wise analysis of the three CNN architectures tested along with the original (not denoised, 20 and 30 s/AFOV acquisitions) scans. Average value ± standard deviation from within the validation set.

| _Image set_ ^Measure^ | MSE  [×10^-3^ g^2^/mL^2^] | SSIM | ICC |
| --- | --- | --- | --- |
| PET20 | 14±5 | 0.91±0.02 | 0.982±0.004 |
| PET20-DL_2.5D_3CAx | 8±3 | 0.93±0.01 | 0.989±0.003 |
| PET20-DL_2.5D | 7±2 | 0.94±0.01 | 0.990±0.002 |
| PET20-DL_3D | 8±3 | 0.94±0.01 | 0.990±0.002 |
| PET30 | 8±3 | 0.94±0.01 | 0.989±0.003 |
| PET30-DL_2.5D_3CAx | 6±2 | 0.94±0.01 | 0.992±0.002 |
| PET30-DL_2.5D | 5±2 | 0.95±0.01 | 0.993±0.002 |
| PET30-DL_3D | 6±2 | 0.95±0.01 | 0.992±0.002 |

MSE: mean squared error; SSIM: structural similarity index measure; ICC: intraclass correlation coefficient; DL_2.5D_3CAx: set denoised through the 2.5D U-Net whose input was each axial slice and the adjacent planes (3 channels); DL_2.5D: set denoised through the 2.5D U-Net; DL_3D: set denoised through the 3D U-Net.

Table S2 – Average coefficient of variation in the liver and lungs for the original (not denoised, 20 and 30 s/AFOV acquisitions) validation images and the DL-denoised validation images through the three methods explored.

| _Image set_ ^ROI^ | CoV_liver_ | CoV_lungs_ |
| --- | --- | --- |
| PET20 | 0.21±0.06 | 0.31±0.09 |
| PET20-DL_2.5D_3CAx | 0.07±0.04 | 0.16±0.09 |
| PET20-DL_2.5D | 0.07±0.06 | 0.17±0.08 |
| PET20-DL_3D | 0.08±0.06 | 0.17±0.09 |
| PET30 | 0.18±0.06 | 0.26±0.07 |
| PET30-DL_2.5D_3CAx | 0.07±0.06 | 0.16±0.08 |
| PET30-DL_2.5D | 0.07±0.06 | 0.16±0.07 |
| PET30-DL_3D | 0.07±0.07 | 0.16±0.08 |

ROI: region of interest; CoV: coefficient of variation; DL_2.5D_3CAx: set denoised through the 2.5D U-Net whose input was each axial slice and the adjacent planes (3 channels); DL_2.5D: set denoised through the 2.5D U-Net; DL_3D: Set denoised through the 3D U-Net.

Table S3 – Normal-uptake organ quantification analysis: average relative difference (± standard deviation) to the reference in SNR and SUV_mean_ in the liver and lungs, from within the validation sets denoised through the 2.5D 3CAx, 2.5D and 3D U-Nets and the original (not denoised) sets.

| ROI | Liver | | Lungs | |
| --- | --- | --- | --- | --- |
| _Image set_ ^Measure^ | ΔSNR [%] | ΔSUV_mean_ [%] | ΔSNR [%] | ΔSUV_mean_ [%] |
| PET20 | -41±20 | -1±4 | -34±15 | +3±10 |
| PET20-DL_2.5D_3CAx | +82±40 | +3±5 | +33±32 | +5±9 |
| PET20-DL_2.5D | +83±40 | +1±4 | +27±29 | +7±8 |
| PET20-DL_3D | +71±38 | +1±3 | +29±33 | +8±9 |
| PET30 | -31±17 | +0±3 | -23±17 | +1±6 |
| PET30-DL_2.5D_3CAx | +86±45 | +3±4 | +39±43 | +3±6 |
| PET30-DL_2.5D | +92±50 | +2±3 | +35±43 | +4±6 |
| PET30-DL_3D | +99±59 | +2±3 | +41±47 | +5±6 |

ROI: region of interest; SNR: signal-to-noise ratio; ΔSNR: SNR variation; SUV_mean_: mean standardised uptake value; ΔSUV_mean_: SUV_mean_ variation; DL_2.5D_3CAx: set denoised through the 2.5D U-Net whose input was each axial slice and the adjacent planes (3 channels); DL_2.5D: set denoised through the 2.5D U-Net; DL_3D: set denoised through the 3D U-Net.

Table S4 – Median absolute deviation of the lesion features (SUV_max_, SUV_mean_, SUV_peak_ and MTV), from within the validation sets denoised through the 2.5D 3CAx, 2.5D and 3D U-Nets and the original (not denoised) sets.

| _Image set_ ^Feature^ | med(\|ΔSUV_max_\|)  [g/mL] | med(\|ΔSUV_mean_\|)  [g/mL] | med(\|ΔSUV_peak_\|)  [g/mL] | med(\|ΔMTV\|)  [cm^3^] |
| --- | --- | --- | --- | --- |
| PET20 | 0.35 | 0.19 | 0.19 | 1.12 |
| PET20-DL_2.5D_3CAx | 0.61 | 0.30 | 0.25 | 0.77 |
| PET20-DL_2.5D | 0.55 | 0.35 | 0.28 | 0.93 |
| PET20-DL_3D | 0.57 | 0.17 | 0.25 | 0.93 |
| PET30 | 0.27 | 0.14 | 0.11 | 0.58 |
| PET30-DL_2.5D_3CAx | 0.39 | 0.18 | 0.21 | 0.45 |
| PET30-DL_2.5D | 0.40 | 0.18 | 0.16 | 0.70 |
| PET30-DL_3D | 0.40 | 0.18 | 0.17 | 0.80 |

SUV: standardised uptake value; SUV_max_: maximum SUV; SUV_mean_: mean SUV; SUV_peak_ – peak SUV; SNR: signal-to-noise ratio; MTV: metabolic tumour volume; med(|Δ$x$|): median absolute variation of variable $x$ (either SUV_max_, SUV_mean_, SUV_peak_ or MTV); DL_2.5D_3CAx: set denoised through the 2.5D U-Net whose input was each axial slice and the adjacent planes (3 channels); DL_2.5D: set denoised through the 2.5D U-Net; DL_3D: set denoised through the 3D U-Net.

Table S5 – Custom score for the different 20-s/AFOV-based validation sets. The images sets were ordered by performance from 1 (worst) to 4 (best), for each measure. Best performance for MSE, CV_liver_, med(|ΔSUV_max_|) and med(|ΔMTV|) corresponded to the lowest value observed, and for SSIM and ICC to the highest value observed.

| _Measure (Weight)_ ^Image Set^ | PET20 | PET20-DL_2.5D_3CAx | PET20-DL_2.5D | PET20-DL_3D |
| --- | --- | --- | --- | --- |
| MSE (10%) | 1 | 2 | 4 | 3 |
| SSIM (10%) | 1 | 2 | 4 | 3 |
| ICC (10%) | 1 | 2 | 4 | 3 |
| CoV_liver_ (25%) | 1 | 4 | 3 | 2 |
| med(\|ΔSUV_max_\|) (30%) | 4 | 1 | 3 | 2 |
| med(\|ΔMTV\|) (15%) | 2 | 4 | 1 | 3 |
| **Weighted score** | 2.05 | 2.50 | **3.00** | 2.45 |

DL_2.5D_3CAx: set denoised through the 2.5D U-Net whose input was each axial slice and the adjacent planes (3 channels); DL_2.5D: set denoised through the 2.5D U-Net; DL_3D: set denoised through the 3D U-Net; MSE: mean squared error; SSIM: structural similarity index measure; ICC: intraclass correlation coefficient; CoV: coefficient of variation; SUV_max_: maximum standardised uptake value; MTV: metabolic tumour volume; med(|Δ$\boldsymbol{x}$|): median absolute variation of variable $\boldsymbol{x}$ (either SUV_max_ or MTV).

Table S6 – Custom score for the different 30-s/AFOV-based validation sets. The images sets were ordered by performance from 1 (worst) to 4 (best), for each measure. Best performance for MSE, CVliver, med(|ΔSUVmax|) and med(|ΔMTV|) corresponded to the lowest value observed, and for SSIM and ICC to the highest value observed.

| _Measure (Weight)_ ^Image Set^ | PET30 | PET30-DL_2.5D_3CAx | PET30-DL_2.5D | PET30-DL_3D |
| --- | --- | --- | --- | --- |
| MSE (10%) | 1 | 2 | 4 | 3 |
| SSIM (10%) | 1 | 2 | 4 | 3 |
| ICC (10%) | 1 | 2 | 4 | 3 |
| CoV_liver_ (25%) | 1 | 2 | 4 | 3 |
| med(\|ΔSUV_max_\|) (30%) | 4 | 2 | 3 | 1 |
| med(\|ΔMTV\|) (15%) | 3 | 4 | 2 | 1 |
| **Weighted score** | 2.20 | 2.30 | **3.40** | 2.10 |

DL_2.5D_3CAx: set denoised through the 2.5D U-Net whose input was each axial slice and the adjacent planes (3 channels); DL_2.5D: set denoised through the 2.5D U-Net; DL_3D: set denoised through the 3D U-Net; MSE: mean squared error; SSIM: structural similarity index measure; ICC: intraclass correlation coefficient; CoV: coefficient of variation; SUV_max_: maximum standardised uptake value; MTV: metabolic tumour volume; med(|Δ$\boldsymbol{x}$|): median absolute variation of variable $\boldsymbol{x}$ (either SUV_max_ or MTV).

## Benchmark Optimisation


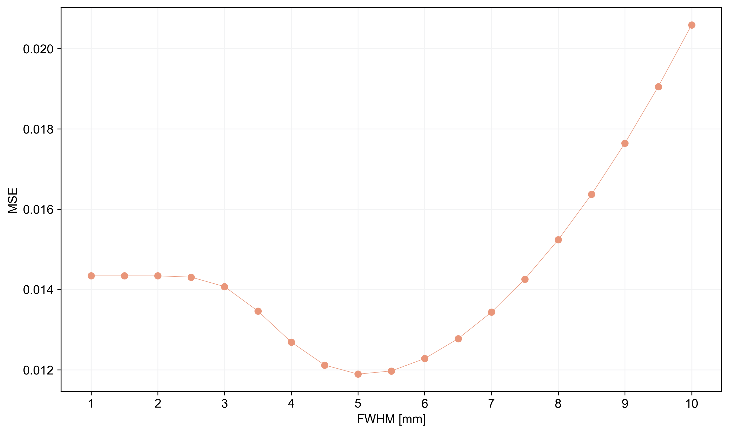


Figure S4 – Gaussian filter optimisation results: minimisation of the MSE between the training set’s fast acquisitions denoised with a specific-width Gaussian filter and respective references. Plot of the average MSE within the training set with respect to filter width.


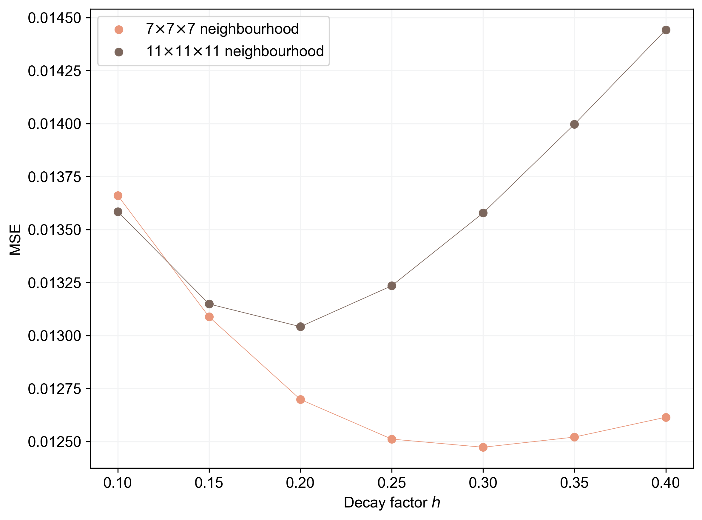


Figure S5 – Non-local means filter optimisation results: minimisation of the MSE between the training set’s fast acquisitions denoised with specific-parameters non-local means filter. Plot of the average MSE within the training set with respect to the decay factor h, for both neighbourhood sizes tested (7×7×7 and 11×11×11).

Figure S5 reveals a parameter-dependent trend in MSE with increasing decay factor *h*. This suggests that the interplay between *h* and the spatial extent of the neighbourhood influences how similar patches are weighted during averaging. At lower *h*, the algorithm overly restricts the averaging to nearly identical patches (between the fixed kernel size and the defined neighbourhood), limiting denoising. At higher *h*, the influence of dissimilar patches increases, potentially introducing bias. At the highest decay factor ℎ = 0.40, the smaller 7x7x7 neighbourhood outperforms the larger 11×11×11 in terms of MSE. This can be attributed to the broader weighting profile induced by higher ℎ, which allows more dissimilar patches to contribute to the filtering. A smaller spatial neighbourhood in this context acts as a constraint, limiting averaging to more locally similar regions and thus counterbalancing the smoothing effect of a larger ℎ. This highlights the parameter-dependent nature of non-local means and its sensitivity to both neighbourhood size and decay rate.
